# Understanding Cellular Redox Homeostasis: A Challenge for Precision Medicine

**DOI:** 10.3390/ijms23010106

**Published:** 2021-12-22

**Authors:** Verena Tretter, Beatrix Hochreiter, Marie Louise Zach, Katharina Krenn, Klaus Ulrich Klein

**Affiliations:** Department of Anesthesia, General Intensive Care and Pain Management, Medical University of Vienna, 1090 Vienna, Austria; trixi.hochreiter@gmx.at (B.H.); marie.zach@gmx.at (M.L.Z.); katharina.krenn@meduniwien.ac.at (K.K.); klaus.klein@meduniwien.ac.at (K.U.K.)

**Keywords:** redox homeostasis, oxidants, antioxidants, precision medicine

## Abstract

Living organisms use a large repertoire of anabolic and catabolic reactions to maintain their physiological body functions, many of which include oxidation and reduction of substrates. The scientific field of redox biology tries to understand how redox homeostasis is regulated and maintained and which mechanisms are derailed in diverse pathological developments of diseases, where oxidative or reductive stress is an issue. The term “oxidative stress” is defined as an imbalance between the generation of oxidants and the local antioxidative defense. Key mediators of oxidative stress are reactive species derived from oxygen, nitrogen, and sulfur that are signal factors at physiological concentrations but can damage cellular macromolecules when they accumulate. However, therapeutical targeting of oxidative stress in disease has proven more difficult than previously expected. Major reasons for this are the very delicate cellular redox systems that differ in the subcellular compartments with regard to their concentrations and depending on the physiological or pathological status of cells and organelles (i.e., circadian rhythm, cell cycle, metabolic need, disease stadium). As reactive species are used as signaling molecules, non-targeted broad-spectrum antioxidants in many cases will fail their therapeutic aim. Precision medicine is called to remedy the situation.

## 1. Introduction

Oxygen is a predominant element on earth and occurs mainly in compound with other elements as oxides. Elementary oxygen (“dioxygen”; O_2_) has become part of the ancient earth’s atmosphere due to the photosynthesis of cyanobacteria. An older form of photosynthesis was anoxygenic and used the then abundant hydrogen sulfide (H_2_S) to reduce carbon dioxide (CO_2_) in order to build biomass. With the appearance of oxygenic cyanobacteria around 2.5 billion years ago, H_2_S and Fe^2+^ became more and more oxidized. Furthermore, atmospheric oxygen has proven to be a highly efficient fuel for early aerobic organisms, that developed aerobic respiration to generate more “adenosine triphosphate” (ATP) compared to anaerobic metabolism. Dioxygen from a physical perspective is relatively inert but is transformed in enzymatic reactions by living organisms to become part of the biomass. Byproducts of such reactions are oxygen radicals which are highly reactive and readily oxidize proteins and lipids and are therefore termed “reactive oxygen species” (ROS) [1]. Low levels of ROS are part of the physiological homeostasis (“oxidative eustress”) and are harmful only at higher concentrations (“oxidative distress”). In order to avoid accumulation of harmful ROS and oxidative distress, cells have evolved cellular antioxidants, which are either enzymes themselves or small molecules, that undergo reversible oxidation. Reduction-oxidation (“redox”) is a key mechanism of life and is essentially a transfer of electrons from a donor (“reducing agent”) to an acceptor (“oxidizing agent”). The term is deferred from oxygen, which is the prototypic oxidizer, but also includes other important elements, that equally engage in redox reactions including nitrogen and sulfur. Cellular redox biology comprises a large network of enzymes, small antioxidants, and reactive species, the status of which is a prerequisite to understand pathological developments and to suggest therapeutic interventions. Reactive oxygen species have attracted most of the research interest of reactive species in the past years, which might be due to a biased view, therefore ROS signaling is better understood than signaling of other reactive species like reactive nitrogen species (RNS) and reactive sulfur species (RSS). 

In a healthy organism, reactive species are messengers, which are regulated and have a high specificity of action. This condition—called “para-hormesis” by Forman and co-workers—ideally allows signaling without tissue damage [2]. This process certainly is not perfect, therefore living organism undergo ageing, which goes hand in hand with the accumulation of oxidatively modified biomolecules. Living organisms are capable to adapt to some extent to an altered redox homeostasis, for instance by upregulating the antioxidative defense (“adaptive homeostasis”). Beyond that, tissue damage occurs, typically in a non-specific way.

In this review we will give a short summary of key players involved in redox homeostasis and their implication in pathological conditions, where oxidative or reductive stress has been identified as part of the disease. The detailed molecular mechanisms of redox stress as known to date have been summarized previously in several excellent reviews, many of which are referenced here. This article aims to address the topic from a translational point of view and to advocate the need of a precision medicine approach for successful therapies.

## 2. Cellular Reactive Species Generation

### 2.1. Reactive Oxygen Species (ROS)

**Figure 1:**
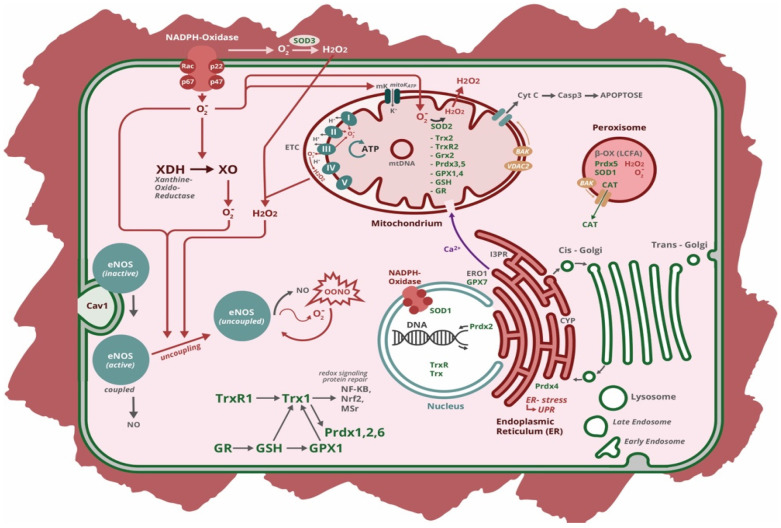
Subcellular generation of reactive oxygen species and the resolving antioxidative defense. The redox potential of redox pairs as well as the concentration of ROS depend on the subcellular compartment. The concentration of H_2_O_2_, for instance, has been determined to be around 80 pM in the cytosol, 20 nM in the mitochondria, 700 nM in the endoplasmatic reticulum and 1–5 µM in the extracellular space (see shades of red). (Abbreviations: NOX: NADPH Oxidase; XDH: xanthine dehydrogenase; XO: xanthine oxidase; SOD: superoxide dismutase; Trx: thioredoxin, TrxR: thioredoxin reductase; GSH: glutathione; GR: glutathione reductase; GPX: glutathione peroxidase; Grx: glutaredoxin; Prdx: peroxiredoxin; eNOS: endothelial nitric oxide synthase; NO: nitric oxide; Cav1: caveolin-1; Ero1: ER oxidoreductin 1; I3PR: inositol-3-phosphate receptor; Cyt C: cytochrome C; Casp3: caspase 3; CAT: catalase; ß-OX: ß-oxidation; LCFA: long chain fatty acid; ETC: electron transport chain; mtDNA: mitochondrial DNA; CYP: cytochrome P450; UPR: unfolded protein response; VDAC: voltage-dependent anion channel; BAK: Bcl-2-homologous antagonist killer protein).

Reactive oxygen species is an umbrella term for various oxygen derived compounds, free radicals, and non-radicals, with highly variable properties including very different rate of reactivity and mode of generation. Main representatives are hydrogen peroxide (H_2_O_2_), superoxide anion (°O_2_^−^), hydroxyl radical (OH°), nitric oxide (NO), peroxynitrite anion (ONOO^−^), singlet oxygen (^1^O_2_), ozone (O_3_), peroxyl radical (ROO°) and hypo-chlorous (-bromous) acid (HOCl, HOBr). Reactive oxygen species are produced either in the course of cell intrinsic biological processes (“intrinsic ROS”) or by extrinsic influence exemplified by radiation, pollutants, xenobiotics, metals and smoke (“extrinsic ROS”). Low levels of intrinsic ROS play an important role in cellular signaling pathways. At these levels, modifications of biomolecules by ROS are reversible switches of protein function and play an important role in cellular processes, such as proliferation, differentiation and migration [3]. Key cellular ROS generating enzymes and subcellular compartments are shown in Figure 1.

#### 2.1.1. Hydrogen Peroxide

H_2_O_2_ is a non-radical ROS, where two oxygens are linked with a single bond. Physiological concentrations lie between 1 and 100 nM. Generation of H_2_O_2_ is mainly triggered by plasma membrane NADPH oxidases (NOX) and by the dissipation of superoxide anion by superoxide dismutases (SODs). H_2_O_2_ as an oxidant has a relatively high specificity towards certain protein cysteine thiols and reacts with Fe-S clusters. It can permeate membranes either directly or by using aquaporin channels and is generally toxic to cells. This is why cells have peroxidase enzymes, that detoxify H_2_O_2_ to water [4]. Hotspots for hydrogen peroxide are peroxisomes (as the name inclines), which are small oxidative organelles. They play an important role in the metabolism of very long chain fatty acids (VLCFA), branched chain fatty acids, bile acid intermediates, D-amino acids, and biosynthesis of plasmalogens. They also contain high amounts of rate-limiting enzymes of the Pentose-Phosphate Pathway (PPP) generating cellular energy and are the location of other organism specific metabolic pathways. H_2_O_2_ is generated there by diverse oxidases and decomposed by catalase.

#### 2.1.2. Superoxide Anion

Superoxide anion (°O_2_^−^) results, when dioxygen (a molecule with two unpaired electrons) accepts one electron to fill one orbital leaving one unpaired electron and a net negative charge. °O_2_^−^ is short lived and transforms spontaneously or enzymatically by SODs to H_2_O_2_, which is more stable. Dependent on pH superoxide is in equilibrium with hydro-peroxyl (HO_2_), which readily diffuses in lipids, although at physiological pH the anion predominates. Phagocytes of the immune system use the superoxide generating enzyme complex NOX at the plasma membrane to kill invading pathogens. NOX-generated °O_2_^−^ at the cytoplasmic side is dismutated by SOD1, while extracellularly released °O_2_^−^ can either re-enter the cell via anion channels (chloride channel-3, CLC-3) or can locally be dismutated by SOD3 to H_2_O_2_, that can re-enter the cell either directly through the plasma membrane or via aquaporin channels. Further, °O_2_^−^ is a byproduct of aerobic respiration at the electron transport chain (ETC) in the inner membrane of the mitochondria, where a small share of electrons “escape” to form °O_2_^−^ from oxygen.

Different types of cells have been shown to respond with a burst of mitochondrial °O_2_^−^ in response to acute moderate hypoxia followed by hypoxic adaptation and metabolic reprogramming including activation of hypoxia-inducible factor (HIF-1) [5,6]. In the cytoplasm, the xanthine oxidoreductase complex is an important source of °O_2_^−^ and H_2_O_2_. In the catabolism of purines, hypoxanthine is oxidized to xanthine and further to uric acid by xanthine oxidase (XO). The enzyme itself is converted from xanthine dehydrogenase (XDH) by oxidation of sulfhydryl residues or proteolytic modification.

#### 2.1.3. Hydroxyl Radical

Hydroxyl radical (°OH^−^) is generated from H_2_O_2_ in the presence of free Fe^2+^ (Fenton reaction). The thereby oxidized iron (Fe^3+^) is reduced to Fe^2+^ by reacting with °O_2_^−^ in a Haber-Weiss reaction and can reenter redox cycling. °OH^−^ is the ROS with highest reactivity and as an unspecific oxidant it reacts directly with the nearest target within a nanometer range (proteins, lipids) at the site of its generation. Therefore, it has a short in vivo half-life (approximately 10^−9^ s). It is a byproduct in some immune reactions and plays a role when immune cells become overactivated and harm nearby otherwise healthy tissues. As such, it has been proven to be a factor in neurological autoimmune diseases but also in neurodegeneration, cardiovascular disease, and cancer. In contrast to other ROS, there are no enzymes that detoxify °OH^−^.

#### 2.1.4. Singlet Molecular Oxygen

The lowest excited form of dioxygen is singlet oxygen (^1^O_2_) with all electrons being spin paired. It is generated by photochemical activation together with some pigments, that thereby for instance induce photosensitivity of the skin (photodermatitis). It also plays a role in the oxidation of LDL cholesterol with resultant cardiovascular effects.

#### 2.1.5. Hypohalous Acids

Hypochlorous acid is known as the active compound of bleach for disinfection. It reacts with -NH2 and -SH groups of proteins, with nucleotides of DNA and RNA, as well as with unsaturated lipids. However, hypochlorous and hypobromous acids also play a role in neutrophils, monocytes, and tissue macrophages. The enzyme myeloperoxidase (MPO) uses H_2_O_2_ to oxidize Cl^−^ ions to hypochlorite in order to oxidize biomolecules, which are further taken up by phagocytes in the course of the resolving phase of inflammation.

#### 2.1.6. ROS in Lipid Peroxidation

Polyunsaturated lipids (PUFAs) are especially prone to oxidation by ROS. Reaction intermediates are the primary lipid peroxidation products lipid peroxyl radicals (LOO°) and hydroperoxides (LOOH), that pertain a kind of chain reaction until antioxidants like vitamin E scavenge the radicals and promote the formation of nonradical end products. Secondary products are malondialdehyde (MDA), propanal, hexanal, and 4-hydroxynonenal (4-HNE), which are used as biomarkers of lipid peroxidation [7,8]. 4-HNE can also function as a signaling molecule of stress pathways as it regulates transcription factors such as nuclear factor erythroid 2-related factor 2 (Nrf2), activating protein-1 (AP-1), nuclear factor ‘kappa-light-chain-enhancer’ of activated B-cells (NF-ĸB) and peroxisome-proliferator-activated receptors (PPAR) [9]. It also activates mitogen-activated protein kinases (MAPK), the epidermal growth factor receptor EGFR/Akt pathway and protein kinase C (PKC). As a result, 4-HNE upregulates heme oxygenase-1 (HO-1), thioredoxin (Trx) and thioredoxin reductase (TrxR) and glutamate cysteine ligase (GCL), the key enzyme in glutathione (GSH) biosynthesis. Furthermore, glycolipids, phospholipids, and cholesterol can be oxidized by enzymes like lipoxygenases, cyclooxygenases, and cytochrome P450 [10].

The unsaturated lipid acid arachidonic acid can be transformed by free radicals to prostaglandin-like compounds called isoprostanes. When membrane phospholipids containing arachidonyl moieties are affected, the membrane function can be severely hampered [11]. Therefore, isoprostanes are rapidly cleaved and released and can be used as biomarkers of oxidative stress in blood or urine.

Lipid hydroperoxides can decompose in vivo through a one-electron reduction, which is a reaction, that further feeds lipid peroxidation by forming peroxyl (LOO°) radicals and aloxyl (LO°) radicals. Alternatively, a two-electron reduction leading to the inhibition of peroxidative damage can be catalyzed by the selenium-containing glutathione peroxidases (GPX) and selenoprotein P (SeP). GPX (located in cytoplasma, nucleus and mitochondria) reduce both, H_2_O_2_ and organic hydroperoxides, to water and the corresponding alcohols and, as the name implies, use glutathione as a reductant. Selenoprotein P is a plasma protein that uses glutathione and thioredoxin as a reductant and protects low-density lipoproteins (LDL) and phospholipids. A specific subtype of the GPX family, GPX4, plays an important role in a distinct iron-dependent type of cell death, called “ferroptosis”. GPX4 oxidizes glutathione to GSSG and thereby reduces cytotoxic lipid peroxides (L-OOH) to the corresponding alcohols (L-OH). Inhibition or degradation of GPX4 leads to the accumulation of lipid peroxides, which facilitates ferroptosis [12].

### 2.2. Reactive Nitrogen Species (RNS)

In 1978 R.F. Furchgott discovered an “endothelium-derived relaxing factor”, later identified as the gaseous substance nitric oxide (NO), which has the capacity to dilate blood vessels. NO is biosynthesized from L-arginine, oxygen and NADPH by essentially three nitric oxide synthases (endothelial, inducible and neuronal NOS). NO is a free radical with an unpaired electron and reacts easily with °O_2_^−^ to form peroxynitrite (ONOO^−^). This reaction depletes the bioactivity of NO thereby affecting smooth muscle tone, blood pressure, platelet activation, and other signaling mechanisms. Peroxynitrite transforms to further reactive nitrogen species, all of which react with and modify lipids, amino acids, nucleotides, and thiols but at a slower rate as compared to the hydroxyl radical. Typical modifications are oxidized cysteines and tyrosine nitration [13].

### 2.3. Reactive Sulfur Species

The central molecule of the cellular sulfur chemistry is the gaseous membrane permeant molecule H_2_S. Sulfur is found just below oxygen in the 6th main group of the periodic table of elements and therefore exhibits similar reaction chemistry. Although evolutionary older, derived RSS have attracted much less attention in the scientific community and the significance of these compounds and derivatives has only been emerging recently. Here, we only briefly describe basic concepts of RSS chemistry, which is rather complex. For more detailed information, we refer the reader to recent comprehensive reviews on this topic [14,15] (and references therein).

H_2_S is generated in different locations of the cytoplasm and in the mitochondria primarily from cysteine and homocysteine, but also from other sources like thiosulfate and carbonyl sulfide. Primary enzymes involved in the synthesis of H_2_S from cysteine are cystathionine γ-lyase (CSE), cystathionine β-synthase (CBS), cysteine aminotransferase (Cat) and 3-mercaptopyruvate sulfur transferase (3-MST). Carbonic anhydrase can produce H_2_S from carbonyl sulfide in the cytosol and D-amino acid oxidase (DAO) can form the intermediate 3-mercaptopyruvate from D-cysteine in peroxisomes, that is further processed by 3-MST in the mitochondria to finally give H_2_S. In analogy to ROS, the transfer of single electrons starting from H_2_S results in the RSS thiyl radical (HS°), hydrogen persulfide (H_2_S_2_) and supersulfide radical (°S_2_^−^) and finally in elemental sulfur. Further complexity is introduced by inter-radical reactions and thiol exchange reactions, that result in the generation of higher order polysulfides (H_2_S_(n)_, RS_(n)_H, RS_(n)_R; R = Cys, GSH, H). It is via these polysulfides, that H_2_S ultimately exerts its effector functions. As such, H_2_S can open ATP-sensitive and voltage-gated potassium channels (K_ATP_, K_v_) and has effects on L-type and Large-conductance Ca^2+^ channels, TRP channels, ion exchangers, and transporters.

Oxidized H_2_S-derivatives can also react with NO to give mixed reactive species like HSNO, SSNO, and ONN(O)-SO_3_^2−^. In the mitochondria cysteinyl tRNA synthase (CARS-2) produces cysteine persulfide (CysSS), which is transported to the cytosol, where CARS-1 forms further Cys polysulfides (CysS_3-6_), that can be simultaneously incorporated into nascent proteins. Interestingly, the ROS detoxifying enzymes SOD_1,2_ and CAT can also oxidize H_2_S to polysulfides.

H_2_S is inactivated by sequential oxidation to sulfate in the mitochondria. Key enzymes for this process are sulfide:quinone oxidoreductase (SQR), the dioxygenase ETHE1, and sulfite oxidase (SO). The resulting electrons are fed into the ETC via cytochrome c (CoQ). When CoQ is already over-reduced, electrons flow backwards to complex I (reverse electron transfer: RET) and leak from there to generate ROS [16,17].

## 3. Cellular Antioxidants

### 3.1. Small Antioxidant Molecules

In 1921, Hopkins discovered the tripeptide -L-glutamyl-L-cysteyl-glycine termed glutathione (GSH), which contains the reducing sulfydryl (-SH) group of the cysteine and is easily oxidized by GPX forming the disulfide-form (GSSG). Reversely, glutathione reductase (GR) can use NADPH to reduce GSSG to GSH. As a key determinant in the defense against oxidative stress, GSH is found in all mammalian tissues (1–10 mM) with the highest concentration in the cytosol (80–85%) and minor amounts in mitochondria (10–15%) and endoplasmic reticulum (<5%). Its biosynthesis is tightly regulated and depends on the availability of the amino acid cysteine [18]. In the liver, cysteine can also be generated by transsulfuration from methionine. Rate-limiting in the synthesis of the tripeptide GSH is glutamate cysteine ligase, which consists of a catalytic (GCLC) and a modifier (GCLM) subunit. The terminal step in GSH synthesis is catalyzed by GSH synthetase. Gene expression of these biosynthetic enzymes is regulated by Nrf2 via the antioxidant response element (ARE) and NF-ĸB. The unusual peptide bond between the γ-carboxyl group of glutamate and L-amino group of cysteine confers resistance to intracellular degradation and can only be cleaved by the extracellular enzyme γ-glutamyltranspeptidase (GGT) as a means of GSH recycling (“γ-glutamyl cycle”). GSH as an antioxidant plays an important role in the dissipation of H_2_O_2_ and regeneration of lipid peroxides by GPX. As a measure and read-out of cellular oxidative stress frequently the GSH/GSSG ratio is used. Glutathione not only cycles between its reduced and oxidized state but can also be linked to thiol groups of proteins (“glutathionylation”) and change protein function or protect these sites from oxidation. This reaction is reversible with the help of glutaredoxin and sulfiredoxin, which also use GSH as a reductant [19].

### 3.2. Vitamins

Vitamin A comprises a group of fat-soluble retinoids and carotenoids including retinol (the storage form of vitamin A), retinal, and provitamin A carotenoids (α, β, γ-carotene), that play important roles in vision, development and immune system function. Structural characteristics are a beta-ionone ring with an attached isoprenoid (retinyl) chain. Vitamin A is a scavenger of hydroxyl radical, superoxide anion, and peroxynitrite and interferes with lipid peroxidation. The antioxidant properties of Vitamin A are only prevalent at a low partial pressure of oxygen (<20 kPa). At higher levels vitamin A becomes a pro-oxidant in autocatalytic reactions.

Vitamin C (ascorbic acid) is an essential vitamin for humans to be taken up from diet, as humans—similar to primates, guinea pigs and some bats—have lost their ability to synthesize this vitamin due to mutations in the biosynthetic enzyme L-gulonolactone oxidase (GULO). A balanced diet leads to plasma concentration of 30–80 µM, which is needed to prevent symptoms of scurvy, such as impaired wound healing, bleeding gums, fatigue, and depression. Primarily, vitamin C acts as antioxidant, as at physiological concentrations, it can reduce ROS resulting in the formation of the oxidation products ascorbate radical and dehydroascorbic acid (DHA). Dehydroascorbic acid can be reduced back to ascorbate with the help of GSH inside the cell or is degraded via 2,3-L-diketoglutonate (2,3-DKG) to oxalic and threonic acid. At higher (mM) concentrations, ascorbic acid can also function as pro-oxidant, which together with its other effects (like effects on iron metabolism and functions as cofactor in enzymatic reactions with metal containing prosthetic groups) are discussed to be helpful in anti-cancer therapy [20].

Vitamin E is a group of lipid-soluble tocopherols and tocotrienols, that have special functions in stabilizing membranes and in protection from lipid peroxidation. Structural details are a common chromane double ring with a hydroxyl group, which can donate a hydrogen ion to reduce free radicals, and methyl groups, whose position determines the subtype. Finally, the hydrophobic side chain of the chromanol ring can insert into biological membranes. Their antioxidant function includes scavenging superoxide and hydroxyl radical and breaking the lipid radical chain reaction.

Bilirubin is an oxidative break-down product of heme in vertebrates. It is generated from biliverdin by a reductase and can again be reoxidized thereby functioning as a cellular antioxidant. In the brain, bilirubin prevents excitotoxicity by scavenging superoxide [21].

### 3.3. Antioxidant Proteins

One of the most important cellular regulators of redox homeostasis is the thioredoxin (Trx) system. Thioredoxins are small proteins that contain two redox-sensitive cysteines in a conserved CXXC amino acid motif, that catalyze the conversion of disulfides into thiols in target proteins, while they become oxidized themselves. The enzyme thioredoxin reductase (TrxR) uses the reducing equivalent NADPH to restore the reduced form of thioredoxin. The thioredoxin system works closely together with the peroxiredoxin (Prdx) family of proteins, which have an important role in the protection from stress conditions. Peroxiredoxins are sensors for H_2_O_2_ and oxidize thiols.

### 3.4. Antioxidant Enzymes

Superoxide dismutases (SODs) catalyze the reaction of superoxide dismutation into O_2_ and H_2_O_2_ mainly at the site of superoxide generation. Enzyme subtypes are confined to compartments, e.g., SOD1 (a Cu/Zn enzyme) is found in the cytosol and the mitochondrial intermembrane space, SOD2 (a Mn enzyme) is localized in the mitochondrial matrix and SOD3 (a Cu/Zn enzyme) is secreted into the extracellular space, where it is partly bound to heparan sulfate polysaccharides of the glycocalyx. The importance of these enzymes is not only the dissipation of superoxide (which is directly harmful for proteins with iron-sulfur clusters, such as enzymes of the Krebs cycle and can also generate ONOO^−^ or induce lipid peroxidation, protein carbonylation and DNA breaks), but also in the generation of H_2_O_2_, which engages in redox signaling [22]. Furthermore, °O_2_^−^ is involved in signaling and has been shown to participate in epigenetic modifications of the DNA and histones (methylation, acetylation) [23].

H_2_O_2_ is detoxified by catalase in the peroxisomes and by peroxiredoxins in the cytoplasm. Peroxiredoxins are peroxidases, that are classified into 3 categories: typical 2-Cys (Prdx1, Prdx2, Prdx3, Prdx4), atypical 2-Cys (Prdx5) and 1-Cys (Prdx6) peroxiredoxins [24,25]. The enzymatic site contains a “peroxidatic” cysteine, that reacts with H_2_O_2_, lipid peroxides or peroxynitrite to form a sulfenic acid. The sulfenic acid reacts with another peroxiredoxin cysteine to form a disulfide bond (an intermolecular bond of typical 2-Cys Prdx and an intramolecular bond of atypical Prdx), which is reduced by thioredoxin. Prdx6 does not form a disulfide, is independent of thioredoxin and uses GSH. Peroxiredoxins are localized to specific compartments: Prdx1, Prdx2, Prdx6 in the cytosol, Prdx3 in mitochondria, Prdx4 in the extracellular space and Prdx5 in mitochondria and peroxisomes. When peroxiredoxins react with two molecules of H_2_O_2_, the enzymes become hyperoxidized (Cys-SOOH) and thereby inactivated. Hyperoxidation can be reversed by sulfiredoxin, which is an ATP-dependent enzyme.

Glutathione peroxidases reduce free H_2_O_2_ as well as lipid hydroperoxides. Again, isoenzymes are located to distinct subcellular compartments and have special preferences for substrates: GPX1: cytoplasm (H_2_O_2_), GPX2: extracellular, intestinal, GPX3: extracellular, plasma; GPX4: lipid hydroperoxides. They are selenoenzymes, and the catalytic reaction comprises oxidation of selenol to selenenic acid, which is reduced again by two molecules GSH. The resulting GSSG is reduced by NADPH in a reaction catalyzed by Glutathione reductase [26]. 

Human Catalase (CAT) is a tetrameric protein with one molecule NADPH bound per subunit and a prosthetic group of ferric protoporphyrin IX. The enzyme is mainly located in the peroxisomes and breaks down H_2_O_2_ to H_2_O and O_2_. Catalase deficiency is associated with several diseases, such as Alzheimer’s and Parkinson’s disease, schizophrenia, diabetes, osteoporosis, and asthma, and is frequently caused by single nucleotide polymorphisms of the CAT gene [27].

### 3.5. Nuclear Factor E2-Related Factor (Nrf2)

Nuclear factor E2-related factor is the central transcription factor of the antioxidant defense and controls more than 200 genes, many of which are antioxidative proteins or involved in detoxification, apoptosis, DNA repair, proteasomal degradation, or regulate inflammation [28]. In the absence of activation signals, Nrf2 is bound to Kelch-like ECH-associated protein 1 (KEAP1) and/or ß-transducin repeat-containing protein (ßTrCP) and is degraded via the proteasome. Oxidative or electrophilic stimuli modify the thiol-rich sensor protein KEAP1 and inhibit GSK3ß, which phosphorylates Nrf2 and facilitates interaction with ßTrCP and thereby dissociates the protein complex. The liberated Nrf2 translocates to the nucleus, binds small musculo-aponeurotic fibrosarcoma (sMaf) or Jun family proteins and interacts with the electrophil response element (EpRE) (or the antioxidant response element -ARE) in the promoter region of target genes, where it is further regulated by interaction with BACH1. Regulation of Nrf2 generally occurs on many levels and is influenced by multiple factors in a gene dependent way. The KEAP1-Nrf2 system maintains crosstalk with the PI3K-AKT signaling system and with autophagy. While Nrf2 knockout mice are viable, KEAP1 knockout mice die before weaning, therefore conditional KEAP1 knockouts were generated to give mechanistic insight in Nrf2 signaling. Nuclear factor E2-related factor null mice revealed enhanced susceptibility to pathology induced by chemicals and xenobiotics (carcinogenesis, acute respiratory distress syndrome, liver toxicity). Diseases associated with oxidative stress, such as ischemia-reperfusion injury, neurodegenerative diseases, hyperoxic lung injury (HALI), asthma, diabetes, rheumatoid arthritis, and sepsis, were exacerbated in these mice. However, excessive activation of Nrf2 (as observed in mouse models with constitutive activation) leads to “reductive stress”, which is due to an overactive NADPH-producing glucose-6-phosphate dehydrogenase and impairs cardiac function (see Section 7).

Nuclear factor E2-related factor also has an important role in dampening and resolving inflammation by modulating the innate immune system. Nuclear factor E2-related factor inhibits the expression of proinflammatory genes, such as the cytokines IL1ß and IL6, and reduces damage-associated molecular patterns. As such, Nrf2 null mice were shown to develop more severe disease progression of emphysema, lipopolysaccharide-induced inflammation, and sepsis as well as multiple sclerosis.

### 3.6. Regulation of Gene Expression of Components of the Cellular Redox Homeostasis

Enzymes producing and neutralizing ROS are tightly regulated in their expression. Swift modulators of mRNA stability are miRNAs, which form an extensive network of regulation, that allow the cell to adapt to the immediate need in redox homeostasis. Many miRNAs in this regulatory system in health and disease have been already experimentally identified [29].

## 4. ROS Generating Systems

### 4.1. Mitochondria

Mitochondria are frequently called the “powerhouse” of the eukaryotic cell, as they produce a large part of the cellular energy in form of ATP. According to the endosymbiotic hypothesis they are believed to be derived from ancient bacteria (Rickettsia type) capable of using oxygen to generate ATP, that were incorporated in eukaryotes as endosymbionts. Indicators for this hypothesis could be their double membrane and the presence of circular chromosomes (mitochondrial DNA, mtDNA), that replicate autonomously. The mtDNA (approximately 16 kilobases) contains genes for redox proteins (respiratory complexes I, III, IV, V), ribosomal RNA (rRNA) and the transfer RNAs (tRNA) [30]. Oxidative damage of mtDNA is repaired by enzymes encoded by the nucleus. However, mtDNA is more susceptible to oxidation of guanines compared to nuclear DNA, as it is not tightly packed by histones.

The outer membrane of the mitochondrion is permeable to molecules smaller than 5 kDa via the channel porin, while the inner membrane is impermeable. Cellular respiration is driven by the electron transport chain (ETC) consisting of five protein complexes in the inner mitochondrial membrane (IMM). In an exergonic process electrons flow from electron donors to acceptors at the same time generating an electrochemical proton gradient across the IMM, that ultimately drives ATP synthesis (“oxidative phosphorylation” at the terminal ATP synthase). Fatty acid oxidation, the citric acid cycle and amino acid oxidation generate the reducing equivalents NADH and FADH_2_, which are the electron donors for the ETC. In the case of aerobic respiration, the final electron acceptor is oxygen, that is reduced by two electrons to water (H_2_O). Leakage of electrons, especially at complexes I and III from the ETC, result in only partial reduction of oxygen to superoxide. °O_2_^−^ is transformed to H_2_O_2_ by the superoxide dismutases SOD2 in the mitochondrial matrix and SOD1 in the mitochondrial intermembrane space. H_2_O_2_ is detoxified by mitochondrial GPX1 and GPX4 and peroxiredoxins Prdx3 and Prdx5. Modified cysteines (glutathionylation and disulphide bonds) are reversed by glutaredoxin Grx2 and thioredoxin/thioredoxin reductase (Trx2/TrxR2). H_2_O_2_ can also diffuse across the mitochondrial membrane into the cytosol. Mitochondrial ROS (mtROS) is not only a byproduct of the ETC, but also has signaling function and amounts rely on cellular energy status. Low levels of mtROS are a means of metabolic adaptation, higher amounts as triggered by danger signals regulate the inflammatory response or can even activate apoptosis and autophagy.

A mitochondrial sensor of cellular oxidative stress is mitoK_ATP_, which is activated by redox-sensitive protein kinase C (PKC) and the resultant K^+^ influx transduces redox signals from the cytosol to the mitochondria by increasing ROS production at the ETC [31,32].

Mitochondria use elaborate quality control pathways, which allow adaptation of cellular stress conditions [33]. This includes regulation of protein importing, protein folding, and the dynamic rate of mitochondrial fusion and fission. In order to avoid propagation of malfunctions, which could lead to a collapse of cellular respiration or excessive ROS generation, irreparable mitochondria undergo a specific from of autophagy, called mitophagy. Mitochondria also play an important role in cellular calcium homeostasis as they take up calcium from the cytosol via the calcium uniporter and release calcium via the mitochondrial sodium/calcium channel. Very high concentrations of calcium together with high levels of ROS can trigger opening of the membrane permeability transition pore (MPT), a complex of the adenine nucleotide translocator, cyclophilin D and the voltage-dependent anion channel (VDAC), followed by release of cytochrome C (Cyt C) and apoptosis.

Mitochondrial free radical generation can be quenched by the endogenous gasotransmitter H_2_S when it is present in low concentrations. Higher amounts of H_2_S, however, are toxic and cause irreversible inhibition of cytochrome oxidase. H_2_S oxidation products enter the ETC via the SQO pathway. Furthermore, H_2_S activates mitoK_ATP_ channels by S-sulfhydration of a cysteine (Cys43 in Kir6.1 regulatory subunit), which stabilizes the channel in an open state [34]. H_2_S further protects against MPT opening, decreases caspase-9 activity, influences mitochondrial biogenesis and cellular contractility and is a vasodilator. These factors contribute to the protective effect of H_2_S on the cardiovascular system under hypoxic conditions and help to alleviate ischemia-reperfusion injury [35]. Therefore, the H_2_S gas itself and H_2_S-releasing drugs are candidates for a new generation of antihypertensive or cardioprotective drugs.

Mitochondria are dynamic structures, that constantly undergo fission and fusion, which allows them to optimize their function and to repair damaged components. Mitochondrial dysfunction underlies several pathologies and diseases and is associated with the ageing process, neurodegeneration, cardiovascular diseases, and heart failure.

### 4.2. Endoplasmic Reticulum (ER)

Proteins, that are to be secreted, enter the ER for correct folding and are then passed on to the Golgi for final glycosylation before they are inserted into the plasma membrane or leave the cell (“=secretory pathway”). Currently, three different ways for a protein to enter the ER are known: SRP-Sec61, GET, or SND-Sec61 pathway. The oxidizing environment in the ER initiates a protein folding process and NXS/T sequences acquire at the same time a precursor N-glycan, that is further trimmed and modified in the Golgi. Protein oxidoreductases mediate disulfide formation in the nascent peptides and misoxidized proteins are concurrently isomerized by respective enzymes (Endoplasmic Reticulum Oxidoreductase-Protein Disulphide Isomerase Pathway: Ero1-PDI) [36]. Terminally misoxidized proteins are prepared for degradation. Ero1 proteins (as flavoproteins) oxidize FADH_2_ to FAD, use the electrons to generate peroxide from O_2_ and together with PDI catalyze the formation of disulphide bonds in the nascent peptide. Peroxide is reoxidized by GPX7/8 and on the other hand Prdx4 uses peroxide to oxidize PDI. The immature oxidized glycoprotein then moves to the calnexin cycle. The oxidative environment in the ER is supported by expression of selenoproteins, as selenocysteines (s) are even more oxidizing than cysteine. In disease states, when misfolded and aggregated proteins accumulate, the Unfolded Protein Response (UPR) is triggered. The UPR sensors are Inositol-requiring transmembran kinase/ endoribonuclease 1α (Ire1α, Protein Kinase RNA-like Endoplasmic Reticulum Kinase (PERK), and Activating Transcription Factor 6 (ATF6) and activate 78-kDa glucose-regulated protein (BiP/grp78) (Hsp 70) induce chaperone expression. Glutathione is the major redox buffer in the ER at double the concentration compared to the cytoplasm (10 mM) [37].

ER and mitochondria are physically and functionally linked including an exchange of ions like calcium and lipids. This is due to mitochondria-associated ER membranes and an ER-mitochondria stress crosstalk. ER-mitochondria contact sites have been implicated as initiation sites for autophagosomes and as crucial sites for metabolic reprogramming in immune cells. The UPR in the ER triggers a metabolic reprogramming of mitochondria towards one-carbon metabolism [38].

### 4.3. Peroxisomes

Peroxisomes are cell organelles enclosed by a single membrane, that contain H_2_O_2_-producing oxidases and catalase. They are specialized in certain metabolic functions, such as catabolism of very long chain fatty acids (VLCFA) by ß-oxidation, biosynthesis of ether-linked glycerolipids (plasmalogens), synthesis of bile acids and metabolism of cholesterol and phytanic acid. In the course of these oxidative metabolic pathways, H_2_O_2_ is generated as a byproduct and is in situ degraded by the local catalase [39]. Catalase is imported into peroxisomes by the receptor Peroxin-5, which recognizes a PTS1 motif typical for peroxisomal matrix proteins. Upon cellular oxidative stress, the otherwise proapoptotic Bcl-2 protein BAK mediates release of catalase into the cytosol as a pro-survival mechanism. Peroxisomes proliferate by growth and division. Membrane assembly and matrix protein import is accomplished by a whole protein family of Peroxins [40]. Defects or deficiency in these proteins lead to a group to Peroxisome Biogenesis Disorders (PBDs), exemplified by Zellweger Syndrome, Neonatal Adrenoleukodystrophy and Heimler Syndrome [41].

### 4.4. NADPH Oxidases

NADPH oxidase (NOX) is special as a family of enzymes, whose primary function is to produce ROS. Superoxide and H_2_O_2_ originating from NOX can affect many cellular components such as nitric oxide synthase (eNOS uncoupling), xanthine oxidoreductase and mitochondria (mtDNA damage, opening of the redox-sensitive mitochondrial ATP sensitive K^+^ channel, mitoK_ATP_). The propagation of ROS originating from NOX to other ROS producing enzymatic systems is called “ROS-dependent ROS production”.

From its initial discovery in phagocytes in the 1960s onwards, seven isoforms of NOX (NOX1-NOX5, DUOX1, DUOX2) have been identified and cloned [42]. NOXs are membrane proteins with multiple transmembrane spanning regions and contain binding sites for NADPH and FAD and 4 histidine residues, that bind heme molecules. The enzymatic activity consists of an electron transfer from NADPH to FAD and via heme to molecular oxygen, which is reduced to superoxide. NOXs are complexes of different subunits. NOX2 for instance has another subunit, p22phox located at the membrane and additionally p40phox, p67phox, and p47phox in the cytosol. Full activation further requires the small GTPase p21-RAC1. Some NOX subtypes additionally have calcium binding motifs (calmodulin binding motifs, EF hands), which allow regulation of their activity by calcium. Expression of different NOX subtypes is cell type specific. Furthermore, subcellular location is dependent on the NOX subtype and cell type and comprises the plasma membrane, endoplasmic reticulum, mitochondria, cytoskeleton, caveolae, nucleus, focal adhesions, and stress fibers. NOX-derived ROS affect many downstream targets including oxidoreductases and the mitochondria. Generated ROS can enter the mitochondria and enhance the mitochondrial ROS production.

### 4.5. Nitric Oxide Synthase

There are two constitutively expressed isoforms of nitric oxide synthase (NOS) in endothelial cells (eNOS) and neurons (nNOS, NOS1) and one inducible NOS (iNOS, NOS2). Under normal conditions, eNOS is a dimer, which is stabilized by tetrahydrobiopterin (H_4_B). The N-terminal oxygenase domain binds L-arginine, H_4_B, heme, and molecular O_2_, while the C-terminal reductase domain binds NADPH. Under physiological conditions, electron transfer is catalyzed from NADPH of monomer-1 to the heme on monomer-2, where O_2_ is reduced and incorporated into the guanidine group of L-arginine with NO and citrulline as final products. The essential factor for proper functioning is H_4_B, which is either synthesized de novo or is retrieved from a salvage pathway (from dihydrobiopterin: H_2_B by dihydrofolate reductase: DHFR). H_4_B deficiency (by DHFR depletion), oxidation of the zinc tetrathiolate cluster at the eNOS dimer, S-glutathionylation of cysteines, however, induce “eNOS uncoupling”. In this case, oxygen is reduced to superoxide, which can react with NO to form peroxynitrite. This phenomenon can occur downstream of NOX activation, as NOX-derived ROS leads to H_4_B deficiency and oxidative modifications (Figure 1). Uncoupling of eNOS is the most investigated as it is implicated in cardiovascular diseases such as diabetes, hypertension, and atherosclerosis [43]. However, nNOS and iNOS can also be uncoupled by similar mechanisms. The renin-angiotensin system (RAS) as an important regulator of blood pressure and cardiovascular function is tightly associated with these ROS generating systems. When endothelial cells are exposed to angiotensin II, it could be shown that NOX-derived ROS (H_2_O_2_) uncouples eNOS (by downregulation of transcription factors regulating DHFR transcription) and thereby reduces the bioavailability of NO (=increased blood pressure) and concomitant further ROS generation. Angiotensin II -induced NOX activation (upregulation of NOX mRNA and protein and AT1 receptor-dependent phosphorylation of p47phox by signaling through Src, PKC and phospholipase C) also increases mitochondrial ROS generation via uncoupling of eNOS and opening of mitoKATP [44]. Uncoupled eNOS produces peroxynitrite, which can damage protein and lipid components of mitochondria. *Vice versa*, mitochondrial ROS also regulate eNOS and NOX, as shown by treatment with antioxidants and inhibitors of mitochondrial ROS generation, that can recouple eNOS and improve endothelial function.

### 4.6. Xanthine Oxidoreductase

The enzyme is synthesized as dehydrogenase (XDH), which is transformed into an oxidase (XO) by oxidation. The name of the enzyme indicates its primary function in purine degradation, namely the conversion of hypoxanthine to xanthine and further to uric acid. XDH uses NAD^+^ as an electron acceptor, whereas XO transfers an electron to oxygen and produces superoxide. XO is a putative factor in certain cardiovascular diseases including hypertension and chronic heart failure. Crosstalk between XO-dependent ROS and mitochondrial dysfunction has been proven experimentally [45].

### 4.7. Cytochrome P-450 System

The Cytochrome P-450 system includes important sites of drug interaction and determinants of individual drug metabolization rates such as CYP3A4 or CYP2D6. Microsomal cytochrome P450 enzymes catalyze the oxygenation of organic substrates (detoxification of xenobiotics, fatty acid oxidation) and thereby reduce molecular oxygen. If the electron transfer is not tightly controlled, the enzyme is uncoupled and ROS are generated [46].

## 5. Redox Regulation in Metabolism

The transfer of electrons is a main reaction type in cellular metabolism including synthesis of amino acids, fatty acids, carbohydrates, and nucleic acids. The redox environment in cellular compartments is equal to the overall reduction potential of all available redox couples. As estimated, the GSSG/GSH or NAD/NADH or NADP/NADPH ratios and read-outs from genetically encoded fluorescence-based biosensors have been frequently used. In order to understand biological systems as a whole organism, systems biology approaches using large data from -omics analysis (transcriptomics, proteomics, metabolomics) have gained high significance in recent years and are also applied to redox biology [47]. Transcriptional profiling, analysis of transcription factor interactions with DNA, analysis of protein expression (including oxidatively modified proteins) and metabolomic profiling are used to analyze physiological cellular processes including pathological developments. These techniques generate a large amount of data that are processed in bioinformatic analyses like network analysis, network inference, and mathematical models to understand ongoing processes on the systems level.

### Redox-Regulated Metabolism in the Immune System

The function of immune cells largely depends on the tightly linked metabolic and redox pathways [48,49] (and references therein). In a resting condition, quiescent T cells use oxidative phosphorylation (OXPHOS) to generate energy from ATP, but upon activation by antigens, they switch from a catabolic to an anabolic metabolism. CD4^+^ and CD8^+^ cells in particular perform aerobic glycolysis with concomitant production of metabolic intermediates, that can be used for the synthesis of amino acids, nucleotides, and fatty acids via the pentose phosphate pathway (PPP), that also generates the reducing equivalents NADPH. The rate-limiting enzyme in nucleotide biosynthesis is ribonucleotide reductase, that generates 2′-deoxyribonucleotides for DNA biosynthesis during rapid proliferation. The reduction reaction is assisted by the thioredoxin and GSH/glutaredoxin system. The thioredoxin system is an essential factor in the anti-tumor responses of CD8^+^ T cells and is under inhibitory control of the negative regulator thioredoxin-interacting protein (TXNIP), which is down-regulated upon T cell stimulation. T cell activation also leads to the import of cystine, which is rapidly reduced to cysteine with the help of thioredoxin and is used together with imported glutamine to generate GSH, the key ROS buffer. GSH controls c-MYC activation, that also releases Trx1 from TXNIP-mediated inhibition. However, low amounts of ROS are important for the activation of nuclear factor of activated T-cells (NFAT) and production of IL-2 thereby facilitating antigen-specific T cell activation as well as for signaling downstream of the T cell receptor (TCR). After T cell stimulation via TCR, the concomitant ROS production induces Nrf2, which counteracts NF-ĸB, activates the Trx/GSH systems, supports metabolic rewiring and is also an important factor in the metabolism of Treg cells. In analogy, B cells also undergo metabolic changes upon activation including fueling of the PPP. B cells generate large amounts of ROS, which is controlled by a highly robust redox system for homeostasis. Antibody production of plasma cells requires disulfide bonds for folding, which is accompanied by ROS formation in the ER. Metabolic rewiring in B cells is facilitated by activation of the PI3K-Akt pathway, that inhibits GSK3, thereby activating c-MYC and mTORC1. Differences in metabolism are also manifested in macrophage differentiation. Dependent on the stimulus, resting macrophages polarize into M1 or M2 phenotype. M1 macrophages use aerobic glycolysis dependent on HIF-1α, PPP, fatty acid synthesis and a truncated TCA cycle with accumulating intermediates to support synthesis of IL-1ß, while M2 macrophages use fatty acid oxidation and mitochondrial oxidative phosphorylation. NADPH as a byproduct of PPP donates electrons to NOX enzymes to kill invading microorganisms and supports the antioxidant systems of Trx and GSH in order to prevent excessive tissue damage. The high amounts of ROS induced by activation of Toll-like receptors (TLRs) induce biosynthesis of GSH, which is necessary for IL1ß mRNA transcription. GSH-dependent GPX4 protects from pyroptosis during sepsis by scavenging lipid peroxides. In the resolution phase, the pro-inflammatory pathways shift to the production of anti-inflammatory metabolites and NADPH is again utilized to support the antioxidant pathways using GSH and Trx.

## 6. Oxidative Stress and Disease

### 6.1. Ageing

With increasing age-free radicals as a byproduct of metabolic processes and their reaction, products accumulate in the organism and play an important role in the ageing process itself (Free radical hypothesis of ageing by D. Harmann). The main location of age-related oxidative damage is the central nervous system (CNS) and especially the brain. Many neurodegenerative diseases have been shown to involve oxidative stress-related pathophysiology. The high energy demand of the CNS is met by using 20% of inhaled O_2_ in mitochondrial OXPHOS, which produces ROS as a by-product. Most exposed to mitochondrial ROS is mtDNA, which is not protected by histones and easily undergoes oxidative damage and mutation. Therefore, mitophagy is an essential correction measure for damaged mitochondria, but this process can also be hampered by oxidative stress as exemplified by S-nitrosylated Parkin in Parkinson’s Disease. DNA damage also activates several kinases and poly (ADP-ribose) polymerase (PARP), which deplete NAD^+^, an essential cofactor in metabolism. These processes induce a vicious cycle of mitochondrial uncoupling, decreased mitophagy and further elevated ROS [50].

### 6.2. Diabetes Mellitus (DM)

Diabetic metabolic disorders comprise the minor collective (approximately 10%) of childhood onset autoimmune disease type I diabetes and the large majority (approximately 90%) of lifestyle-dependent later onset type II diabetes. Type II diabetes (T2D) prevalence has increased significantly in the last decades especially in wealthy countries and is predicted to further increase in the coming years in an epidemic-like manner, thereby representing a significant burden to the healthcare systems. T2D represents a heterogenous disease, where different metabolic shifts lead to hyperglycemia, increased insulin resistance and later insulin secretion deficiency by islet ß-cells [51]. Chronic hyperglycemia leads to non-enzymatic modification of proteins (“glycation products”) such as hemoglobin A1c (HbA1c), which is routinely used as a long-term biomarker for glucose levels in DM patients. Protein glycation and oxidative degradation of glycated proteins as well as glucose oxidation is accompanied by formation of free radicals and a decline in the antioxidant defense mechanisms. Diagnostic read-outs are changes in oxidative stress biomarkers such as superoxide dismutases, catalase, glutathione and glutathione reductases and peroxidases, lipid oxidation products, and nitrite.

Glucose is physiologically metabolized in the body initially by glycolysis ending up with the terminal product pyruvate, which is decarboxylated and enters the Krebs cycle in the mitochondria activated by coenzyme A. The Krebs Cycle produces reducing equivalents NADH and FADH_2_ used by the ETC for the proton gradient. While under physiological conditions the ETC produces small amounts of ROS, this is highly increased under hyperglycemic conditions as in DM. The resultant oxidative stress induces damage of biomolecules including DNA, that activates the repair enzyme poly-ADP-ribose polymerase-1 (PARP-1), which is an inhibitor of a key enzyme in glycolysis, glyceraldehyde-3-phosphate dehydrogenase (GAPDH) and therefore induces accumulation of upstream glycolysis intermediates including glucose itself. These glycolysis intermediates can undergo auto-oxidation or fuel other pro-oxidative pathways such as hexosamine and polyol pathways as well as advanced glycation end-product (AGE) and PKC pathways [52]. An overactive hexosamine pathway leads to higher levels of UDP-GlcNAc increasing O-glucosamine-N-Acetyl transferase (OGT) activity that regulates gene expression. Furthermore, proliferation of the collagen matrix and basement thickening are caused by this metabolic shift. The normally minor polyol pathway gains in significance under hyperglycemic conditions and then consumes large amounts of NADPH, which is also an important cofactor of GSH-dependent antioxidant enzymes. PKC activity stimulates NADPH oxidases and lipooxygenases, thereby elevating the cellular ROS burden.

The increasing oxidative stress triggers a vicious cycle of further ROS generation and compromises the regulation of blood glucose levels by insulin. Hyperglycemia-induced oxidative stress has an impact on insulin secretion from pancreatic ß-cells mediated by ATP-dependent K^+^ channels, that become uncoupled under these conditions. Oxidative stress also impairs the translocation of insulin-stimulated glucose transporter 4 (GLUT4) to the cell surface and impairs phosphorylation in the insulin receptor signaling cascade.

Generally, the role of oxidative stress in DM is multi-faceted and dysregulations of metabolic pathways affect many different levels, which makes therapeutic interventions extremely difficult.

### 6.3. Neurodegeneration-Alzheimer’s Disease

Many of the neurodegenerative diseases are age-related and are frequently associated with oxidative damage of enzymes involved in CNS metabolism. Hallmark of Alzheimer’s Disease (AD) is deposition of senile plaques (fibrillary amyloid-ß peptide) and neurofibrillary tangles (hyper-phosphorylated tau protein). AD has been also called “Diabetes type 3”, as glucose metabolism is impaired and brain insulin resistance is observed. Furthermore, T2D is a significant risk factor to develop AD. Redox proteomics has shown, that in brains with AD some enzymes of glucose metabolism are preferentially oxidatively damaged, such as GAPDH, phosphoglycerate mutase, enolase, fructose biphosphatase aldolase, triose phosphate isomerase, pyruvate dehydrogenase, and aconitase [53,54]. Typical oxidative stress markers are protein carbonyls, glycosidated proteins (AGE formation), protein-coupled HNE, 3-nitrotyrosine and DNA damage marker 8-hydroxy-deoxyguanosine (8-OHdG), and lipid oxidation products F2/4-isoprostanes. Dysfunctional mitochondria are also typical for the disease, with defects in sirtuin 3 and ATP synthase. Additionally, activation of mTOR inhibits autophagy and leads to insulin resistance in analogy to T2D. Escape from autophagy deficiency to the ubiquitin-proteasome system (UPS) is also hampered due to oxidative deactivation of involved enzymes. Typically, markers of ER stress are upregulated in AD brains and different deregulated pathways ultimately lead to neuronal cell death. Preclinical studies have given evidence, that antioxidant therapies might be helpful, but clinical trials with vitamin E were rather disappointing, because the vitamin might not reach the brain in sufficient concentrations. Polyphenols like resveratrol and quercetin have shown some promise [55]. These compounds are slightly pro-oxidant and work by inducing upregulation of the body’s own antioxidative defense. A similar paradoxon is observed with the enzyme heme-oxygenase (HO-1). HO-1 is an oxidant of heme, but the end-product bilirubin is a ROS scavenger. Interestingly, some statins including atorvastatin have been shown to upregulate HO-1 and extended intake has been proven to result in a lower risk of developing AD [56].

### 6.4. Cancer

Many types of cancer develop from or go hand in hand with a chronic state of inflammation and a high level of ROS. Involved inflammatory cells further propagate ROS by activation of enzymes like NOX, XO, iNOS, COX2 and lipoxygenase (LOX). As a response, cancerous cells upregulate their antioxidative defense (frequently by activation of Nrf2 transcription factor), which prolongs their survival and makes attempts to therapeutically attack them more difficult. The regulation of oxidative stress and levels of reactive oxygen species is important in both the development of tumor cells as well as their response to treatments. For many years those treatments aimed solemnly at the regulation of genes involved in tumorigenesis (“oncogene addiction”) [57]. Newer approaches in anti-cancer therapies, however, also target the metabolism and immune surveillance fueling these cells [58]. Combatting ROS in cancer can be a two-edged sword: it might decrease ROS-related cell damage (including DNA), but it might also further increase the tumor cells’ anyhow strong antioxidative capacity. At initially lower levels, ROS play a key role in tumor development, as they act as signaling molecules for proliferation and differentiation, are involved in the activation of stress-response pathways and might drive mutation in affected cells. Reactive oxygen species signaling is for instance implicated in epithelial-mesenchymal transition (EMT), a key process in cancer development. This includes ROS activation of TGF-ß activated Smad, p38 and ERK MAPkinases, Snail, E-cadherin and integrins, hepatocyte growth factor receptor HGFR/c-Met, Ap-1 and Ets-1 activation. Furthermore, signaling involved in metastasis is largely ROS-dependent, such as Wnt/T-cell factor (TCF), diverse kinases and phosphatases. Reactive oxygen species, in addition to hypoxic conditions and growth factors, also enhance VEGF levels that are involved in tumor associated angiogenesis. Excessive levels of ROS, however, can also cause damage and drive cell death; therefore, several studies have been performed to determine where the threshold from an oncogenic to a tumor suppressive effect of ROS lies. Tumor cells frequently exhibit an altered redox balance, which can provide resistance to oxidative stress inducers given for therapy. An increased resistance to oxidative stress allows tumor cells to increase their metabolic rate and to further enhance their proliferation without getting damaged by associated ROS generation. Some newer anti-cancer drugs operate by inducing excessive oxidative stress by generation of ROS and reducing cellular antioxidants. Examples are the platinum complexes such as cisplatin and anthracyclines, such as doxorubicin. Additionally, PARP inhibitors enhance DNA damage and are tested in ovarian and breast cancer. Some of the chemotherapeutics are combined with drugs, that modulate GSH levels at the same time, in order to support the cytotoxic effects of ROS generation by lowering GSH at the same time. Furthermore, Trx inhibitors are used to target the other strong antioxidative system [59]. 

A growth advantage of tumor cells is also their alterations in metabolism, especially glucose metabolism. Cancer cells frequently switch their metabolism to aerobic glycolysis (Warburg effect), which allows a high glycolytic rate accompanied by low ROS generation avoiding cellular senescence. Therapeutic approaches to target this special property of cancer cells use glycolysis inhibitors such as non-hydrolysable glucose (2-deoxyglucose) or glycolytic enzyme inhibitors. Some of these drugs have been successfully used in preclinical tests and also clinical trials [60].

### 6.5. Ischemia-Reperfusion Injury

Ischemia-reperfusion injury can occur when tissues experience a period of hypoperfusion followed by reperfusion. During ischemia, metabolism is shifted towards an anaerobic mode producing less ATP and antioxidants with concomitant acidosis and failure of ion pumps. Reperfusion generally is lifesaving but also generates large amounts of ROS with their detrimental consequences including endothelial dysfunction, protein and DNA damage, and inflammation. Major sources of ROS during reperfusion are the xanthine oxidoreductase system, the ETC, uncoupled NOS, and NOX enzymes [61]. In severe cases, cells can undergo apoptosis, necrosis, or other forms of cell death. Therapeutical approaches try to target the main factors in the pathogenesis, such as reducing the duration of ischemia, correcting acidosis, using anti-inflammatory treatments, or pharmacological inhibition of ROS producing systems such as xanthine oxidase. A preemptive measure in organ transplantation is the technique of ischemic preconditioning [62].

### 6.6. Lung Diseases

The Berlin Definition of Acute Respiratory Distress Syndrome (ARDS) from 2012 classifies the severity of ARDS according to a decreased arterial blood oxygen tension (PaO_2_) relative to the inspired fraction of oxygen (FiO_2_) in steps of 100 mmHg from mild (201–300 mmHg) to moderate (101–200 mmHg) to severe (<100 mmHg) ARDS. Typical for the disease is a loss of barrier function of the lung capillary endothelium and alveolar epithelium leading to edema and inflammation as shown by an increase in inflammatory markers and neutrophil migration into the tissue. Plasma and bronchoalveolar lavage fluid (BALF) biomarkers are IL-6, IL-8, surfactant protein-D, receptor for advanced glycation end products (RAGE), and club cell secretory protein (CC-16) [63].

ROS are involved in the impairment of the endothelial barrier, because caveolin-1 (transcellular pathway) and junctional proteins (paracellular pathway) are affected with regard to their expression and function, which is often regulated by phosphorylation. The lung is also the organ with the highest expression of components of the renin-angiotensin system. The metabolite Angiotensin II enhances ROS and thereby triggers endothelial permeability. The paracellular pathway is regulated by adherens junctions (AJs) made up by cadherins/catenins including VE-cadherin and tight junctions (TJs) are composed of claudins, occludins, and junctional adhesion molecules (JAMs). A well described mechanism of barrier disruption is the enhanced endocytosis of VE-cadherin due to an increased level of intracellular phosphorylation, which is normally counter-regulated by a protein tyrosine phosphatase (PTP). However, PTPs are sensitive to oxidation, therefore ROS can lead to increased VE-cadherin phosphorylation and resultant endocytosis. Additionally, ROS can decrease expression of TJ proteins and affect oligomerization of occludins, which depends on the GSH/GSSG ratio. Modifications of the actin cytoskeleton leads to the formation of stress fibers, that expand inter-endothelial gaps. Reactive oxygen species also affect intracellular calcium fluxes and down-stream enzymatic activities, which all have an impact on barrier function. Polymorphonuclear cells (PMNs) transmigrate the endothelium via the transcellular and paracellular route using cell surface adhesion molecules (CAMs) including selectins and integrins as well as Ig superfamily members ICAM and VCAM. Expression of CAMs is regulated by the transcription factors NF-ĸB and AP-1, both of which are regulated by ROS [64].

Therapies targeting the redox systems showed mixed results. A proposed adjunct treatment for patients with ARDS is N-acetyl cysteine (NAC), an antioxidant which can be used by the body as a precursor of glutathione. Preclinical data showed a reduction of pro-inflammatory cytokines and indicated beneficial effects in lung injury due to hyperoxia and LPS. In clinical studies, it has been shown that NAC improves respiratory function but does not enhance the survival rate; however, it did not show adverse effects [65]. Inhaled NO transiently improved oxygenation due to vasodilation, but also had no positive effect on mortality [66,67]. A therapeutical potential of the gases H_2_S and CO has been discussed [68,69]. Despite their toxicity in larger concentrations, these gases are metabolites in the conversion of L-cysteine and heme oxygenase, respectively, and are attributed protective effects. Furthermore, in animal experiments, NOX inhibitors such as apocynin and Nrf2 activators have shown beneficial effects [70]. Activated Protein C (APC) is a zymogen, that interferes with thrombin generation. Activated protein C is anti-inflammatory and anti-apoptotic and stabilizes barrier function. However, in clinical studies it did not excel over the saline control group [71].

Chronic obstructive pulmonary disease (COPD) is characterized by a progressive chronic bronchitis and emphysema, that leads to an increasingly obstructive pattern in pulmonary function tests. Triggers for its development include external oxidants from cigarette smoke or air pollution. Disease progression is accompanied by additional ROS/RNS generation from endogenous sources like NOX, myeloperoxidase (MPO) or iNOS. Multiple antioxidant therapies have been tested for treatment of COPD in addition to bronchodilators and corticosteroids. Examples are recombinant human Trx and NAC with some success especially in preclinical studies [72].

Cystic fibrosis (CF) is a progressive genetic disease caused by mutations in the cystic fibrosis transmembrane conductance regulator (CFTR). Hallmarks of the disease are a massive recruitment of neutrophils to the airways releasing proteases and oxidants and at the same time a deficiency of glutathione, which normally is the major antioxidant in the epithelial lining fluid [73]. Supplementation of glutathione by inhaled or oral doses of NAC is long-standing practice, but with limited success. More recent therapeutical approaches try to directly target neutrophil-derived damaging molecules, such as hypochlorous acid or MPO [74].

Asthma is an inflammatory disease of the airways (bronchi, bronchioles) and is caused by environmental and/or genetic factors. Oxidative stress is induced by elevated ROS/RNS and a decrease in the antioxidative defense including SODs, CAT and GPXs. Exhaled breath condensates of asthmatic patients frequently contain elevated levels of NO, H_2_O_2_ and 8-isoprostanes. However, clinical studies using antioxidant therapies have not shown any clear benefit [75].

### 6.7. Viral Infection-SARS CoV-2

Viral infections are frequently accompanied by neutrophil infiltration, concomitant ROS generation and a decrease in antioxidant defenses. Respiratory viral infections such as coronavirus infectious disease (COVID)-19 are associated with an inhibition of Nrf2 pathways and NF-ĸB-dependent activation of inflammation and oxidative damage. Many COVID-19 patients have reduced serum thiol levels and decreased availability of H_2_S indicating redox imbalances [76,77]. Increased amounts of reactive species are generated from mitochondrial dysfunction and enhanced signaling of angiotensin II via AT1-receptors. The redox state of cysteines in the SARS-CoV-2 spike protein and in the host cell receptor ACE2 seems to be important for the interaction of these proteins. A disulfide bond between Cys-133 und Cys-141 of ACE2 at the dimer interface seems to be crucial for host cell entry [78]. The homologous amino acid of Cys-133 in cattle and swine is a leucine, which might be a reason, why these species are resistant to SARS-CoV-2.

COVID-19 patients frequently reveal strong neutrophil infiltration in pulmonary capillaries with increased circulating neutrophil extracellular traps (NETs) and nucleotide-binding oligomerization domain-like receptor containing pyrin domain 3 (NLRP3) inflammasome activation creating a cytokine storm. Excessive neutrophilia leads to large amounts of ROS damaging pulmonary and blood cells thereby generating a pro-coagulant state with dysfunctional red blood cells and final hypoxic respiratory failure [79]. Antioxidants especially from food sources have also been proposed as supplemental therapy [80].

### 6.8. Critical Illness

Critical illness as treated in intensive care units (ICUs) comprises different clinical pictures including sepsis, ARDS, multiorgan failure and cardiogenic shock. In some cases, such as sepsis, large amounts of reactive species are generated due to the overactivation of phagocytic cells, in addition to an inflammatory state. Mitochondrial function is also frequently impaired in critical illness, sometimes as a result of oxidative damage to mitochondrial membranes by lipid peroxidation. Dying cells also release the mitochondrial components mtDNAs, which in their function as DAMPs can activate immune processes as they contain bacteria-like characteristics (unmethylated CpG, formylated peptides) [81]. Additionally, mtDNA can initiate the NLPR3 inflammasome, activate TLR9, and was shown to be a predictor of mortality in some cases [82,83].

Organ failure in sepsis is mediated by macro- and microcirculatory abnormalities including vascular permeability and mitochondrial free radical generation. These factors contribute to septic shock, activation of cytokine production from liver Kupffer cells and apoptotic cardiac dysfunction. GSH can improve vascular endothelial dysfunction, however, peroxynitrite is detrimental [84]. ETC deficiency and increased mitochondrial calcium levels are frequently observed in patients with poor prognosis [85].

Assessment of oxidative stress in critically ill patients is sometimes difficult, as this might be tissue- or organ specific and depends on the interplay of pro-oxidants and antioxidant capacity. Pro-oxidants are estimated by the measurable by-products such as MDA, isoprostanes or 8-OHdG and the antioxidant capacity can be assessed as the capability of a plasma sample to scavenge an in vitro generated free radical. Selenium is important as a cofactor of more than 30 selenoproteins including antioxidant enzymes such as s SeP, GPX family proteins and TrxR, and plasma levels have frequently been shown to be reduced in ICU patients. Therefore, selenium supplementation via the parenteral or enteral route has been proposed and tested in several clinical studies; however, the benefit with regard to reduced mortality rates have not been unambiguously proven [86]. Zinc is another important cofactor, as it increases activity of GPX, SOD and CAT enzymes, upregulates Nrf2 and inhibits NOX, iNOS, NF-κB and NMDA receptors. Further, it binds to sulfhydryl groups protecting proteins from oxidation and competes with iron and copper and thereby inhibits radical formation at cell membranes and concomitant lipid peroxidation. Data from clinical trials in ICU patients solely focusing on zinc supplementation are scarce, but show a tendency toward lower mortality [87,88].

Vitamin C has also been used as an adjunct in sepsis therapy. A systematic review and meta-analysis indicated a trend towards a reduction in short-term mortality in patients receiving vitamin C as additional intervention [89].

## 7. Reductive Stress and Disease

If the physiological equilibrium is perturbed in such a way, that cellular redox couples (i.e., NAD/NADH, NADP/NADPH; GSSG/GSH, FAD/FADH_2_) make an unhealthy shift to their reduced form exceeding the capacity of oxidoreductases, signaling pathways can equally be affected in a detrimental way resulting in “reductive stress”, that goes hand in hand with disturbance of physiological ROS signaling, perturbation of protein-thiol modifications, ER stress and metabolic reprogramming. Paradoxically, reductive stress can also increase ROS production, for instance by one-electron transfers to oxygen, activation of NOX enzymes or via ETC complexes I and IV [90]. The interconversion of NAD(P)/NAD(P)H is a concomitant of cellular metabolism, in particular glycolysis, pentose phosphate pathway and the tricarboxylic acid cycle [91]. Subcellular compartments have their own pool of NAD(P)(H), which reflects the local situation and exchange between compartments is mostly restricted to shuttles and transporters (malate/aspartate shuttle, glycerol-3-phosphate shuttle, isocitrate-α-ketoglutarate shuttle). Similarly, the GSSG/GSH equilibrium is also dependent on the subcellular compartment with a large excess of GSH in cytoplasm and mitochondria, but with almost equal concentrations of GSH and GSSG in the ER. Inter-compartmental exchange again is performed by carriers. Apart from determining the setpoint of the redox potential of this redox couple and thereby setting the redox tone of the compartment, glutathione also engages in protein modifications (S-glutathionylation), which determines structure and function of the respective proteins.

### 7.1. Cardiovascular Disease

A disturbed redox balance can manifest itself in different forms of cardiomyopathies. A frequently discussed example in context of reductive stress is a protein-aggregation cardiomyopathy induced by mutations in a chaperone of desmin (αB-crystallin), which plays a crucial role in the contractibility of myofibrils. Mutated mouse models revealed a state of reductive stress including upregulation of antioxidant enzymes (GPX1) via Nrf2 activation and proteins involved in NADPH generation (glucose-6-phosphate dehydrogenase; G6PD) [92]. Crossing these animals to mice with reduced G6PD expression alleviated the phenotype of cardiac hypertrophy. Furthermore, inhibiting GPX1 in mice with increased GPX1 expression improves the hypertrophic phenotype. Upregulation of Nrf2 activity possibly occurs via ER stress, which is mediated via Ire1, PERK and ATF6. Especially PERK can phosphorylate Nrf2 and thereby disrupts the inhibitory complex with KEAP1.

The importance of a balanced redox state in the heart is also revealed by transgenic mice overexpressing NOX4 or knock-down mice. However, the results from different heart-failure models gave an unclear picture of mechanisms behind the observed phenotypes [93].

### 7.2. Diabetes Mellitus

Diabetic hyperglycemia initially leads to an accumulation of NADH similar to a hypoxic condition, where oxidative phosphorylation is tuned down and anaerobic glycolysis is increased. This “pseudohypoxic” increase in NADH/NAD ratio results in reductive stress with concomitant mitochondrial dysfunction and excess ROS generation. In diabetes NADH is produced by glycolysis and the polyol pathways and an overactivation of PARP, which uses NAD^+^ [94]. As a consequence, NAD^+^− dependent enzymes, such as sirtuin lysine-deacetylase (Sirt1) are restricted in their activity. Sirt1 is involved in mitochondrial biogenesis.

Signaling induced by insulin, like also other growth factors, relies on a certain amount of ROS, which keep protein tyrosine phosphatases at bay and thereby keep kinases active. NOX enzymes have been shown to play an important role in this aspect [95]. Mouse models overexpressing antioxidant enzymes have revealed that loss of ROS attenuates intracellular signaling induced by insulin, which infers a state of insulin resistance resembling type 2 diabetes [96,97].

## 8. Antioxidants in Therapy—What Are the Problems?

As oxidative stress has been identified as the primary cause of disease or as secondary accompanying phenomenon in the progression of many other diseases, it was an obvious attempt to use antioxidants for therapy. In the 1960s, the Nobel laureate Linus Pauling developed his hypothesis, that high doses of vitamin C should protect from many diseases as exemplified by influenza, schizophrenia, and cancer [98]. Despite his otherwise groundbreaking works, this hypothesis is highly controversial and has evoked concern on the one hand and admiration on the other hand among scientists, physicians, and nutritionists. Many clinical studies using dietary vitamin C supplementation have not shown any benefit. The picture of vitamin C therapy has only recently been modified by further research. Meanwhile, distinct mechanisms are known to explain why especially high-dose intravenous vitamin C therapy could be a safe and effective means of inhibiting cancer growth [20]. Ngo et al. propose that this therapy might be especially suitable for certain cancer patient subpopulations with defined mutations in cancer-associated genes, indicating a special task of -omics technology in precision medicine [20].

N-acetyl cysteine is a water-soluble antioxidant, that is taken up via an anion exchange protein and deacetylated intracellularly to release the amino acid cysteine, a building block for GSH. It is used for its mucolytic properties to treat respiratory conditions, cystic fibrosis and intoxication with paracetamol. Results from experimental and clinical studies in the context of other diseases however are conflicting [99]. Another way to replenish GSH levels is GSH itself that needs to be esterified to be resistant to degradation by plasma γ-glutamyl transpeptidase and to be able to transverse cell membranes due to their lipophilic nature. Inside the cell, the esters are cleaved by non-specific esterases and yield GSH. Experimental evidence is available to show that these GSH esters are able to increase GSH levels in different organs, but clinical studies are still lacking [100].

To enhance antioxidative enzymes a possible approach is to either use individual enzymes or their mimics or in a more complex way increase their expression by activating the transcription factor Nrf2. Mimetics of SOD include the metalloporphyrins, Mn-salen complexes, Mn cyclic polyamines, and nitroxides [101]. Some of the mimetics show extended reactivity towards other ROS and in some cases have also pro-oxidative properties in certain context. The most promising data are derived from preclinical studies and some drugs have entered early human clinical phases to prove their safety. The best known GPX mimetic is the compound 2-phenyl-1,2-benzisoselenazol-3(2H)-one alias epselen [102]. Epselen has been tested in several clinical studies including support of treatment of Meniere’s disease, bipolar disorder, and ischemic stroke and has shown positive properties. Nrf2 activators have promising effects in diseases, where the innate Nrf2 signaling is compromised or dysregulated [103,104]. Due to the multiple mechanisms of Nrf2 activity regulation there are different drug targets. Examples are dimethyl fumarate (approved by the Food and Drug Administration and Pharmaceuticals and Medical Devices Agency for patients with multiple sclerosis), quercetin (in clinical trials for COPD, autism, hepatitis C, COVID-19, etc.), resveratrol (in clinical trials for dilated cardiomyopathy, diabetes, cystic fibrosis, inflammatory bowel disease, etc.), curcumin (in clinical trials for schizophrenia, depression, AD, atherosclerosis, cervical cancer, etc.), and sulforaphane from broccoli (in clinical trials for COPD, depression, diabetes, autism etc.). Inhibitors of BACH1 have been suggested for older patients, as other Nrf2 activators have been shown to be less efficient in this group of patients [105]. 

Some cancers show persistent activation of Nrf2, which affects drug resistance and the proliferation rate [106]. In these cases, Nrf2 antagonism might be implicated. Some Nrf2 inhibitors, such as brusatol or halofuginone have been shown to sensitize cancer cells to chemo- and radiotherapy; however, these drugs require a suitable delivery system directly to the cancerous tissue in order to do no harm to healthy organs and tissues. Another drawback might be, that Nrf2 inhibitors are expected to activate suppressor cells leading to enhanced cancer metastasis [107].

Excessive oxidative stress can occur by overactivation of NOX enzymes generating large amounts of °O_2_^−^. Therefore, NOX inhibitors, that either target the active site or prevent NOX subunit assembly like ebselen and apocynin have been used in clinical trials [108].

Several diseases exhibit elevated ROS levels, and in many cases, this can be referred to mitochondrial dysfunction. Therefore, mitochondria-targeted antioxidants became a new line of therapeutical research [109]. An argument in favor of this approach is that mitochondrial ROS would be scavenged at the locus of their generation without disturbing ROS signaling in other parts of the cell. Examples for such drugs are MitoQ (a derivative of ubiquinone conjugated to triphenyl phosphonium, which allows the drug to enter and accumulate within the mitochondria), SKQ1 (plastoquinonyl decyltriphenyl phosphonium), Mito-TEMPO, and MitoVitE. However, intervention studies have not unequivocally proven beneficial effects, some even revealed harmful effects [110]. Key problems are different methodologies, lack of specificity, achieving an effective concentration at the target site, and unspecific actions of antioxidants.

Regarding the likeliness of success of antioxidant therapy, Forman and Zhang point out that a main factor is to distinguish, whether oxidative stress is a primary causal factor of disease, or a secondary concomitant factor, which occurs in the course of pathological developments [2]. If oxidative stress is primary, meaning a disease triggering factor, scavenging reactive species with antioxidants might be more likely to be successful regarding therapy outcome. However, this will occur only in a minority of cases. If oxidative stress is only a secondary factor (=in most cases), treatment with antioxidants might alleviate some symptoms, but effects might only remain minor.

Many clinical trials have been conducted that used easily available untargeted antioxidants, such as glutathione, N-acetyl cysteine, vitamin C, curcumin, or resveratrol as a diet supplement for treatment of diseases, where oxidative stress had been shown to be a confounder. However, many of these studies were unsuccessful, either because the therapeutic antioxidants lacked specificity with regard to the involved reactive species, the subcellular compartment or the organ/tissue or other obstacles such as a wrong timing and dosage, or unfavorable bioavailability and metabolism. A neglected issue might be also that high doses of antioxidants could in some cases induce reductive stress, that -as discussed earlier-can by itself induce ROS generation. In order to develop successful therapies, a detailed understanding of molecular events in disease development and progression is a desirable prerequisite and targeted therapies will be in most cases superior compared to broad spectrum untargeted drugs with regard to outcome. Therefore the precision medicine approach seems to be appropriate to increase the success rate of therapies in diseases concomitant redox stress (Table 1).

An emerging field of research is antioxidant gene therapy, which has been used in animal models of disease states with success in several cases. This approach comprises the use of viral vectors, such as adenovirus, lentivirus or adeno-associated viral (AAV), receptor mediated gene delivery or non-viral carriers, such as cationic molecules (small molecules, lipids and peptides), engineered exosomes or polymeric micelles and modified nanoparticles [111]. The implementation of nanotechnologies has provided tools to improve drug physical properties and stability inside the body and to specifically target the drugs to a target tissue [112,113]. In addition, gene editing therapy can make use of the CRISP/Cas9 gene editing technique to modify individual proteins, such as KEAP1 in [114]. 

However, overarching expectations from single antioxidant therapy probably would need to be put into perspective, as oxidative/reductive stress frequently is not the original cause of the disease, but only a bystander. Furthermore, trying to restore an individual dysbalance might not be suitable to fix the highly complex inter-dependent network of other changes in the redox systems.

## 9. Perspectives

As translational medicine makes progress in understanding disease initiation, development, and progression, new approaches can be undertaken to address such complex problems like those observed in dysregulated redox homeostasis. Precision medicine is not only a hopeful treatment option in malignant cancers, but will certainly gain broader importance in other fields including all redox biology, opening up “Precision Redox”, a term introduced by Meng et al. [115]. This will include understanding acute and chronic reactive species signaling in health and disease, oxidative modifications of macromolecules as identified by -omics techniques and developing suitable targeted drugs or switchable genetic modifications to specifically address pathological changes in the redox balance. Many of these approaches have already been tested with significant success in vitro and in animal models. However, as always, the next steps to clinical trials in humans are a big challenge of the future.

## Figures and Tables

**Table 1:** Precision Medicine in Diagnosis and Therapy of Diseases associated with Redox Stress.

**Diagnosis**			
	**clinics**	Antioxidant/Oxidative Stress status	
		Biomarker	Oxidative stress markers, ox. mtDNA, extracellular thiols Precursors of antioxidants and reactive species (glycine, homocysteine, arginine) and co-factors (folate, Vitamin B_6_, B_12_)
		Tumor profiling	Tissue proteomics, cfDNA, Exosomes
	**-omics**	Genomics	GWAS, SNPs
		Transcriptomics	Microarrays, RNA sequencing, mRNA, miRNA (NGS)
		Proteomics	Redox proteomics
		(Redox-)Metabolomics Systems Biology Approaches	*Challenge:* individual differences, cohort heterogeneity, demographic, environmental, dietary influence, identification of independent functional disease markers
**Therapy**			
		Drug dosage, formulation, targeting	
		Genome editing/engineering	CRISPR/Cas9, TALENs, ZFNs

Examples of methodological approaches in diagnostic and therapeutical precision medicine (Abbreviations: ox. mtDNA: oxidated mitochondrial DNA; cfDNA: circulating free DNA; GWAS: genome-wide association studies; SNPs: small nuclear polymorphisms; miRNA: microRNA; NGS: next generation sequencing; CRISPR/Cas9: clustered regularly interspaced short palindromic repeats/CRISPR-associated protein 9; TALENs: transcription activator-like effector nucleases; ZFNs: zinc finger nucleases).

## Data Availability

Not applicable.
